# Single-center experience with robotic colorectal surgery for benign indications

**DOI:** 10.1007/s00384-026-05179-7

**Published:** 2026-07-02

**Authors:** Sowmiya Gunabalasingam, Krupa George, Chamaldi de Silva, Selena Tsz Wai Chang, Fang Yi Cheung, Valentin Butnari, Phakanant Chaichanavichkij, Sandeep Kaul, Matthew Hanson, Richard Boulton, Joseph Huang, Nirooshun Rajendran

**Affiliations:** 1 Department of Surgery, Havering and Redbridge University NHS Trust, BarkingLondon, UK; 2 The Centre for Neuroscience, Surgery and Trauma, Faculty of Medicine and Dentistry, Blizard Institute, Queen Mary University of London, National Bowel Research Centre, Abernethy Building, 2 Newark Street, London, E1 2AT UK

**Keywords:** Robotic surgical procedures, Prior abdominal surgery, Colorectal surgery, Colectomy, Surgical outcomes

## Abstract

**Purpose:**

Robotic colorectal surgery is established for malignant indications, but evidence supporting its use in benign disease remains limited. We describe perioperative outcomes of 403 consecutive robotic colorectal resections across all indications at a single UK NHS center, with primary focus on safety across benign indications.

**Methods:**

Retrospective analysis of a prospectively maintained database (February 2020-November 2025). Cases were stratified by indication: malignant or highly suspected malignancy (*n* = 332, 82.4%) and benign (*n* = 71, 17.6%). Benign cases were subclassified into diverticular disease (*n* = 39), ulcerative colitis (*n* = 14), Crohn’s disease (*n* = 13), and other (*n* = 5). Primary outcomes were operative time, length of stay, and Clavien–Dindo complication grade.

**Results:**

Benign patients were younger (median 53 vs. 68 years; *p* < 0.001) with higher rates of prior abdominal surgery (33.8% vs 19.6%; *p* = 0.012). Across all 403 cases, median operative time was 240 min (IQR 199-302), median LOS 5 days (IQR 4-8), and CD Grade III-IV rate 9.2%. Despite equivalent operative times (*p* = 0.84), benign cases converted more frequently (15.5% vs 6.9%; *p* = 0.031), independently predicted by age (OR 1.04; *p* = 0.013), and cancer indication (OR 0.31; *p* = 0.012). Within the benign cohort, IBD patients were younger than those with diverticular disease (*p* < 0.001). Procedure type and stoma rates differed significantly by subgroup (both *p* < 0.001), yet complication rates, operative time, and LOS were equivalent.

**Conclusion:**

Robotic colorectal surgery is safe and reproducible across malignant and benign indications within specialised colorectal robotic centers. Complication profiles matched published benchmarks. Benign cases had a higher conversion rate, but this was driven by prior surgical burden and operative complexity, not platform limitation, and it did not increase morbidity. These data constitute the largest single-center UK evidence base for robotic colorectal surgery across all indications and support the expansion of robotic commissioning into benign practice.

## Introduction

The landscape of surgical practice has been significantly transformed since the US Food and Drug Administration’s approval of the first surgical robot for clinical use in 1994 [1]. Since then, robotic technology has rapidly evolved, becoming a preferred modality across numerous surgical specialities like urology, gynecology, thoracic, and gastrointestinal surgery [2, 3]. With the new robotic systems and platforms, a trend is noticed towards increasing utilisation and expanding application across all specialities [4–6].

Since the first robotic-assisted colectomy was performed in 2002 for diverticulitis, robotic approach gained in popularity in well-developed countries, becoming the main approach in many specialised colorectal centers [7, 8]. Compared to conventional open and laparoscopic approaches, robotic surgery offers distinct advantages, including stable three-dimensional visualisation and enhanced range of movements [9]. These features contribute to improved surgical precision, leading to better patient outcomes such as reduced intraoperative blood loss and shorter hospital stay [10]. Although robotics has multiple advantages within the field of colorectal surgery, it also comes with certain disadvantages, including high cost of the platforms, consumables, servicing, and the lack of tactile feedback [11].

Robotic-assisted surgery has been integrated into the treatment of both malignant and benign colorectal condition conditions. Its enhanced precision and wristed articulation 540 degrees rotation and 7 axes of motion compared to surgical methods, particularly for procedures in confined areas like the pelvis. This has led to a surge in the popularity of robotic platforms for colorectal surgery, especially demonstrating promising short- and long-term oncological outcomes as well as functionally with improved urogenital and sexual outcomes in rectal cancer treatment [12–14]. Despite the substantial body of evidence primarily supports its efficacy in colorectal malignancy, there is comparatively less data published on its applicability in surgical treatment of benign disease.

Patients suffering from benign colorectal conditions like inflammatory bowel disease (IBD), particularly those with Crohn’s disease and ulcerative colitis, often need multiple surgeries throughout their lives [15]. This also applies to complicated diverticular disease, which may initially be managed with interventional radiology but often requires subsequent resection, with the use of robotic and laparoscopic approaches for elective sigmoid resection for diverticular disease showing comparable operative and perioperative outcomes [16]. A major hurdle for colorectal surgeons in these cases is the presence of advanced inflammatory process or adhesions from previous abdominal surgeries [17]. These adhesions distort normal anatomy, making the initial steps of the surgery, such as inducting of pneumoperitoneum and placing trocars potentially challenging. However, once the ports are placed, robotic surgery offers distinct advantages. It facilitates adhesiolysis and provides a more ergonomic experience for the surgeon. However, to foster broader implementation of robotic-assisted surgeries for nonmalignant colorectal conditions, more robust data and a higher level of evidence are essential to support its feasibility [18].

Primarily, this retrospective cohort study aims to present our institutional experience within the use of the robotic platform for the surgical treatment of benign colorectal conditions compared to malignancy.

## Materials and methods

### Study design and setting

A single-center retrospective observational study was undertaken to evaluate robotic surgery in surgical treatment of oncologic and benign colorectal disease treatment. This was undertaken between February 2020 to November 2025 at our Trust (a large district general Hospital in outer North East London). All surgeries were performed by 5 consultant colorectal surgeons but included our learning curve as well. All procedures were performed using the da Vinci Xi platform with table motion. The surgical techniques and ports setup were previously described in the literature [14]. This study has been reported in line with the Preferred Reporting Of Case Series in Surgery (PROCESS) 2023 guidelines criteria [19].

### Patient selection

All consecutive patients undergoing robotic colorectal resection within the study period were eligible for inclusion. Cases were classified by primary operative indication as follows:

Cancer/malignancy-resections for confirmed malignancy or lesions with suspicious histopathological features without conclusive invasion, but incongruent radiological features for benign disease.

Benign-all other indications. Benign cases were further subclassified based on preoperative diagnosis: diverticular disease; ulcerative colitis; Crohn’s disease; or other (comprising sigmoid volvulus, colonic angiodysplasia, fecal incontinence with prolapse, and familial adenomatous polyposis).

Emergency cases requiring immediate laparotomy without robotic platform deployment were excluded.

Patients lost to 30-day follow-up were also excluded. An initial screening log of all robotic cases performed within the trust for the audit period was done. Data was collected based on the operative theater lists from electronic healthcare records.

### Variable and outcomes

We collected the following demographic variables: age, body mass index (BMI), and ethnicity.

Clinical data consisted of WHO performance status [20], the American Society of Anaesthesiologists (ASA) classification [21], type of the operation, stoma formation total operative time, intraoperative estimated blood loss in milliliters, and conversions were captured.

Postoperative outcomes included length of stay, length of intensive care unit stay, and the rate of 30-day complication rate as well as 30-day readmission rate.

The primary outcome of interest was the 30-day complication rate classified as per the Clavien-Dindo classification system [22]. The secondary outcomes included length of stay (LOS), and 30-day readmission rate.

Total operative time defined as the duration in minutes from the first surgical incision to the completion of skin closure.

Conversion was defined as the decision to abandon the robotic approach intraoperatively, requiring a laparotomy with a midline incision > 5 cm to complete the procedure.

Anastomotic leak was defined as any communication between intra- or extraluminal compartments at the site of the anastomosis confirmed either surgically or radiologically.

Intra-abdominal collection was defined as a postoperative fluid accumulation confirmed by imaging or requiring drainage intervention.

Surgical site infection was defined as an infection within abdominal wall structures, in the port site incision or extraction site.

Prolonged ileus was defined as the presence of abdominal distension, intolerance to oral intake, and delayed passage of flatus or stool beyond postoperative day 3.

>Bleeding intraluminal bleeding that bleeds into the gastrointestinal tract or extraluminal bleeding that occurs into the abdominal cavity.

Patient comorbidity was quantified using the Charlson Comorbidity Index (CCI), a validated scoring system that assigns weighted points to 17 medical conditions and age category, with higher scores predicting greater 10-year mortality risk. CCI was calculated from the following recorded fields: diabetes mellitus (1 point), chronic pulmonary disease (COPD or asthma, 1 point each), congestive heart failure (1 point), end-stage renal disease (2 points), and history of prior solid malignancy (2 points). For cancer cases, the primary tumor was scored as a solid malignancy (2 points). Age contributed 0 points (below 50 years), 1 point (50 to 59 years), 2 points (60 to 69 years), 3 points (70 to 79 years), or 4 points (80 years or above). Fields not available in the dataset (myocardial infarction, peripheral vascular disease, cerebrovascular disease, dementia, peptic ulcer disease, mild or severe liver disease, and hemiplegia) were scored as zero; this represents a systematic underestimation that is acknowledged in the limitations. For analysis, patients were classified as CCI below 6 (low to moderate comorbidity burden) or CCI 6 or above (high comorbidity burden, associated with predicted 10-year survival below 10%).

### Research ethics review

Institutional ethics committee review did not require ethical approval for this study. This study was a service delivery evaluation of the use of robotic platform in surgical treatment of benign colorectal conditions. Patient consent was not required. The study was conducted in accordance with the Declaration of Helsinki (as revised in 2013).

### Data analysis

Continuous variables were assessed for normality using the Shapiro-Wilk test (*n* ≤ 50) or D’Agostino-Pearson omnibus test (*n* > 50). Given that the majority of continuous variables did not follow a normal distribution, results are presented as median (interquartile range, IQR). Two-group comparisons of continuous variables used the Mann-Whitney *U* test; comparisons across three or more groups used the Kruskal-Wallis *H* test. Categorical variables are reported as frequency (*n*) and percentage (%); comparisons used Fisher’s exact test (2 × 2 tables or expected cell frequency < 5) or Pearson’s chi-square test as appropriate. A multivariable binary logistic regression model was constructed to identify independent predictors of conversion to open surgery, with candidate predictors selected on the basis of clinical relevance and univariable association (*p* < 0.10). Results are expressed as odds ratios (OR) with 95% confidence intervals (CI). All tests were two-sided; *p* < 0.05 was considered statistically significant. Analyses were performed using R studio.

## Results

**Fig. 1:**
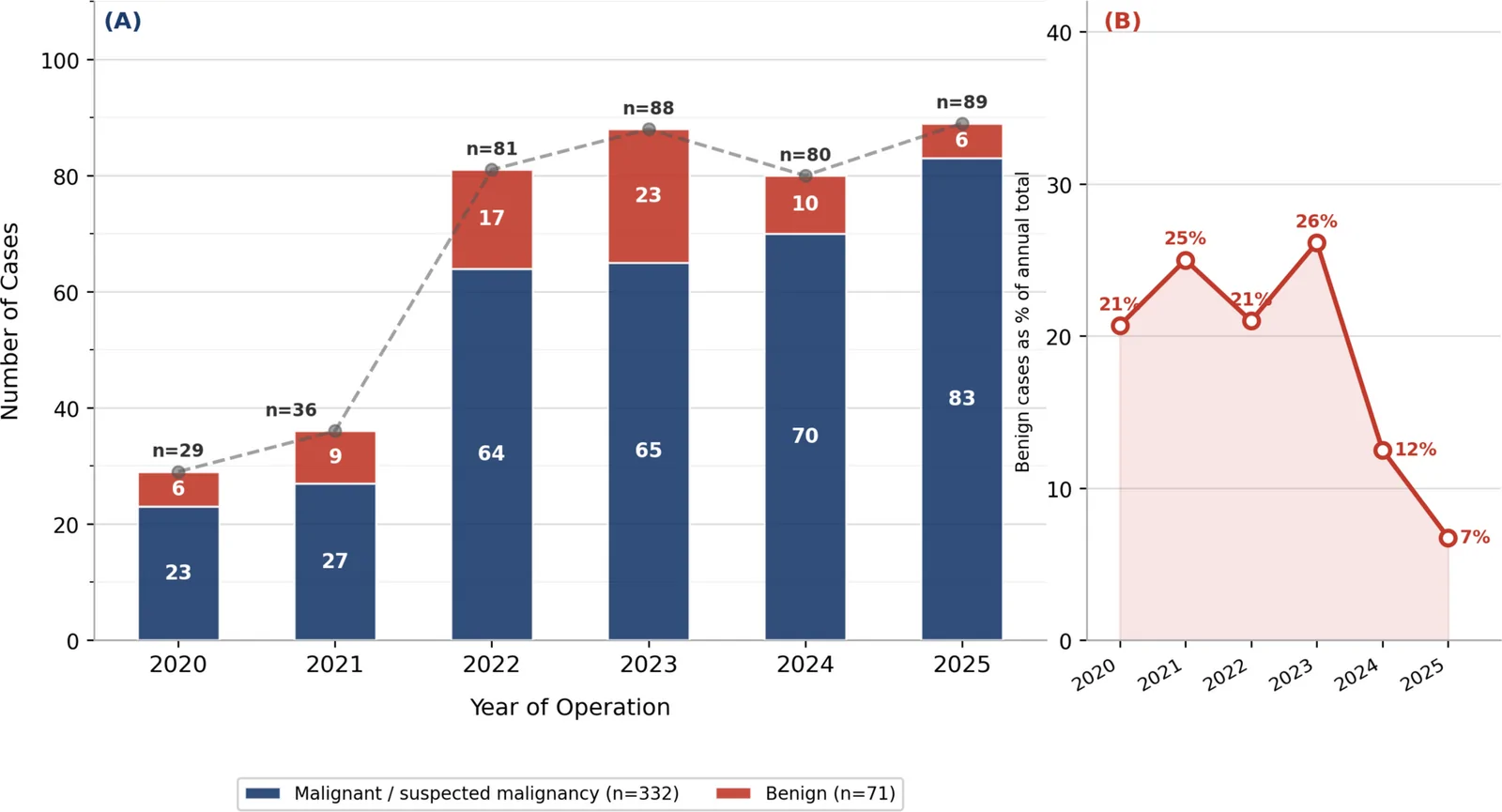
Annual operative by indication. **A** Stacked bar chart showing the number of robotic colorectal resections performed each year, stratified by indication. **B** Benign cases expressed as a percentage of the total annual programme volume

Four hundred and three consecutive robotic colorectal resections were performed between February 2020 and November 2025 across five consultant surgeons. Three hundred and thirty-two cases (82.4%) were performed for malignant or highly suspected malignancy indications and 71 (17.6%) for benign pathology (Fig. 1). Cancer patients were significantly older median 68 vs 53 years; *p* < 0.001 and ASA grade distribution was not significantly different between groups (*p* = 0.471). Prior abdominal surgery was significantly more prevalent in benign patients (33.8% vs 19.6%; *p* = 0.012), reflecting staged IBD operative complexity. Charlson Comorbidity Index (CCI), cancer patients had a median CCI of 5 (IQR 4 to 6) versus 1 (IQR 0 to 2) in the benign group (Mann–Whitney *U*, *p *< 0.001). CCI 6 or above was present in 124 of 332 cancer patients (37.3%) and in only 2 of 71 benign patients (2.8%, Fisher’s exact *p* < 0.001). Total cohort outcomes are presented (Table 1).

**Table 1:** Demographic and perioperative outcomes of total cohort

Variable	Cancer (*n* = 332)	Benign (*n* = 71)	*p*-value
Demographics			
Age, years median (IQR)	68 (58-76)	53 (40-67)	< 0.001***
Male sex, *n* (%)	176 (53.0%)	30 (42.3%)	0.117
BMI, kg/m^2^ median (IQR)	27.7 (24.3-31.0)	26.3 (23.3-31.0)	0.348
ASA 3–4, *n* (%)	107 (32.2%)	17 (23.9%)	0.471
Prior abdomino-pelvic surgery, *n* (%)	65 (19.6%)	24 (33.8%)	0.012*
CCI median (IQR)	5 (4 to 6)	1 (0 to 2)	*p* < 0.001
CCI below 6, *n* (%)	208 (62.7%)	69 (97.2%)	< 0.001
CCI 6 or above, *n* (%)	124 (37.3%)	2 (2.8%)	< 0.001
Intraoperative			
Operative time, min median (IQR)	240 (200-300)	240 (190-320)	0.841
Estimated blood loss, mL median (IQR)	50 (50-50)	50 (50-100)	0.014*
Conversion to open, *n* (%)	23 (6.9%)	11 (15.5%)	0.031*
I TU stay, nights median (IQR)	1 (0-1)	0 (0-1)	0.010**
Stoma formed, *n* (%)	66 (19.9%)	23 (32.4%)	0.027*
Recovery and postoperative outcomes			
LOS, days median (IQR)	5 (4-8)	6 (4-10)	0.089
Overall complications, *n* (%)	79 (23.8%)	18 (25.4%)	0.761
Anastomotic leak, *n* (%)	16 (4.8%)	3 (4.2%)	1.000
SSI, *n* (%)	19 (5.7%)	8 (11.3%)	0.113
Intra-abdominal collection, *n* (%)	18 (5.4%)	8 (11.3%)	0.104
Prolonged ileus, *n* (%)	28 (8.4%)	8 (11.3%)	0.491
CD Grade III-IV, *n* (%)	28 (8.4%)	9 (12.7%)	0.261
30-day readmission, *n* (%)	24 (7.2%)	1 (1.4%)	0.099

^*^p < 0.05; **p < 0.01; and ***p < 0.001

Mann–Whitney *U* test (continuous); Fisher's exact test or chi-square (categorical). Significant *p*-values highlighted red

*SSI* = surgical site infection; *CD* = Clavien–Dindo; *LOS* = length of stay; *ITU *= intensive therapy unit

Despite benign cases being undertaken in a significantly younger cohort (median age 53 vs. 68 years), total operative time was statistically equivalent (median 240 min; *p* = 0.84). Benign cases demonstrated a significantly higher conversion rate to open surgery (15.5% vs 6.9%; *p* = 0.031; Table 1). Multivariable logistic regression identified age (OR 1.04; 95% CI 1.01-1.08; *p* = 0.013) and cancer indication, which was protective against conversion (OR 0.31; 95% CI 0.13-0.77; *p* = 0.012), as independent predictors of conversion. Cancer patients had significantly higher intraoperative blood loss (*p* = 0.014) and ITU utilization (*p* = 0.010), reflecting the older age and comorbidity burden in this group rather than greater operative complexity. LOS was one day longer in benign cases (median 6 vs. 5 days) but this trend did not reach significance (*p* = 0.089). Operative and recovery outcomes are illustrated (Table 1).

Overall complication rates were statistically equivalent between groups (cancer 23.8% vs. benign 25.4%; *p* = 0.76). No individual complication category reached significance, including anastomotic leak (4.8% vs. 4.2%; *p* = 1.00), SSI (5.7% vs. 11.3%; *p* = 0.11), prolonged ileus (8.4% vs. 11.3%; *p* = 0.49), CD Grade III-IV events (8.4% vs. 12.7%; *p* = 0.26), and 30-day readmission (7.2% vs. 1.4%; *p* = 0.10).

**Fig. 2:**
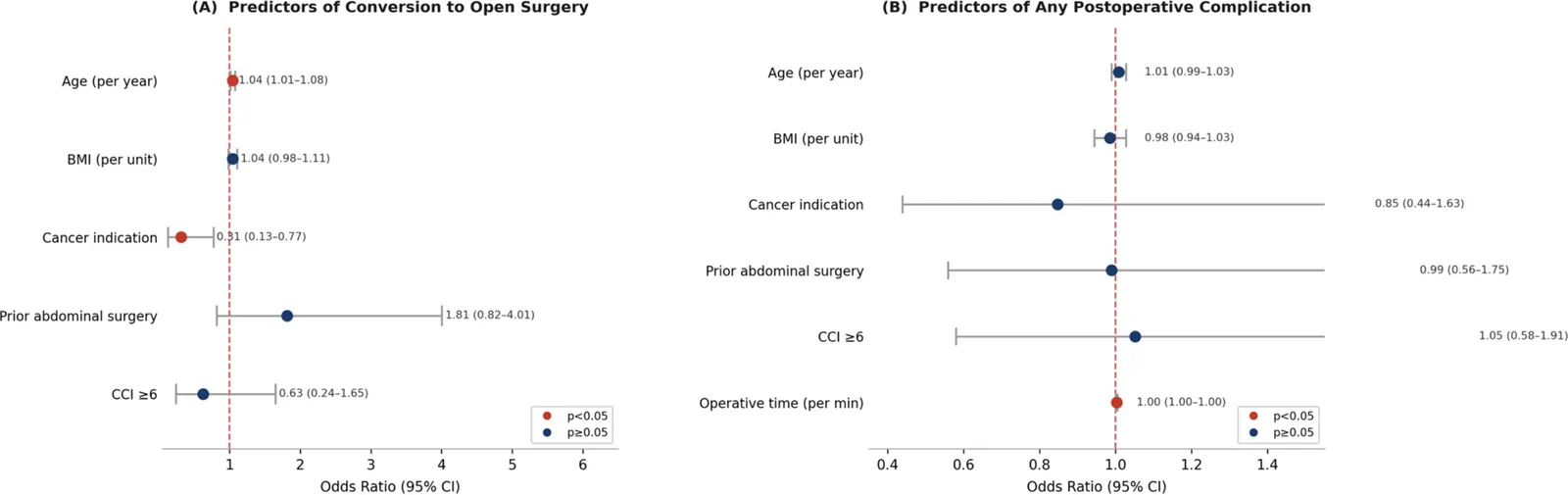
**A** Forest plot with independent predictors of conversion to open surgery. **B** Forest plot representing independent predictors of any postoperative complication

Multivariable logistic regression (Fig. 2) identified longer operative time (OR 1.003; 95% CI 1.000-1.005; *p* = 0.019) as the sole independent predictor of any postoperative complication. Benign vs. malignant indication, BMI, estimated blood loss, stoma formation, and prior abdominal surgery were not significant independent predictors. The lower readmission rate in the benign cohort (1.4%) reflects the predominantly younger, lower-comorbidity patient profile.

The cancer cohort comprised 332 cases. Right-sided tumors (*n* = 98, 29.8%) included the appendix (*n* = 9, 2.7%), ascending colon/hepatic flexure (*n* = 48, 14.6%), cecum (*n* = 41, 12.5%), and transverse colon (*n* = 23, 7.0%). Left-sided tumors were more prevalent, consisting primarily of the sigmoid/rectosigmoid (*n* = 81, 24.6%), and the splenic flexure/descending colon (*n* = 21, 6.4%). The remaining cases involved the and the rectum (*n* = 49, 14.9%) and anal canal (*n* = 5, 1.5%). R0 resection was achieved in 96.6% (258/267 evaluable cases), a figure consistent with international registry benchmarks for robotic and laparoscopic colorectal resection. Median lymph node yield was 20 (IQR 15–26); 93.6% of evaluable cases yielded ≥ 12 nodes, meeting the minimum recommended by ACPGBI guidance.

The benign cohort (*n* = 71) comprised diverticular disease (*n* = 39, 54.9%), UC (*n* = 14, 19.7%), Crohn’s disease (*n* = 13, 18.3%), and other benign pathology (*n* = 5, 7.0%). The five cases in the “other” subgroup comprised sigmoid volvulus, colonic angiodysplasia, fecal incontinence with prolapse, and FAP. Given this heterogeneity and small group size, results for this subgroup are reported descriptively only. The most clinically significant inter-subgroup difference was age (Kruskal–Wallis *p* < 0.001). Patients with Crohn’s disease were younger (median 34 years, IQR 26-43), followed by those with UC (median 40 years, IQR 24-55); both were substantially younger than the diverticular disease cohort (median 61 years, IQR 52-69). BMI, sex distribution, and ASA grade were statistically equivalent across subgroups (*p* = 0.13, *p* = 0.81, and *p* = 0.46, respectively). Comorbidity burden, measured by the Charlson CCI, also differed across subgroups (Kruskal–Wallis *p* < 0.001). Diverticular disease patients had the highest median CCI at 2 (IQR 1 to 3), consistent with their older age profile and associated medical conditions. UC and Crohn's disease patients had a median CCI of 0 (IQR 0 to 1), reflecting their younger age and lower chronic disease burden. CCI 6 or above was rare in the benign cohort overall: 2 of 39 diverticular disease patients (5.1%) and none of the IBD patients.

Prior abdominal surgery was most prevalent in UC patients (64.3%). This borderline significant difference (*p* = 0.059) likely reflects insufficient statistical power given the subgroup sizes rather than a true null effect. Subgroup demographics are presented (Table 2).

**Table 2:** Baseline demographics by benign subgroup

Variable	UC (*n* = 14)	Crohn’s *(n* = 13)	Diverticular (*n* = 39)	Other (*n* = 5)	*p*-value
Age, years median (IQR)	40 (24-55)	34 (26-43)	61 (52-69)	48 (37-61)	< 0.001***
BMI, kg/m^2^ median (IQR)	23.2 (21-28)	30.1 (24-33)	26.3 (25-31)	25.8 (21-28)	0.134
Male sex, *n *(%)	6 (42.9%)	7 (53.8%)	15 (38.5%)	2 (40.0%)	0.811
ASA 1, *n* (%)	2 (14.3%)	2 (15.4%)	1 (2.6%)	1 (20.0%)	0.457
ASA 2,* n* (%)	11 (78.6%)	8 (61.5%)	25 (64.1%)	4 (80.0%)	
ASA 3, *n* (%)	1 (7.1%)	3 (23.1%)	12 (30.8%)	0 (0.0%)	
Prior abdominal surgery, *n* (%)	9 (64.3%)	4 (30.8%)	10 (25.6%)	1 (20.0%)	0.059
CCI median (IQR)	0 (0 to 1)	0 (0 to 1)	2 (1 to 3)	0 (0 to 2)	< 0.001***
CCI below 6 *N* (%)	14 (100%)	13 (100%)	37 (94.9%)	5 (100%)	0.686
CCI 6 or above *N* (%)	0 (0%)	0 (0%)	2 (5.1%)	0 (0%)	

***p < 0.001

Kruskal-Wallis *H* (continuous variables); chi-square or Fisher’s exact (categorical variables)

**Fig. 3:**
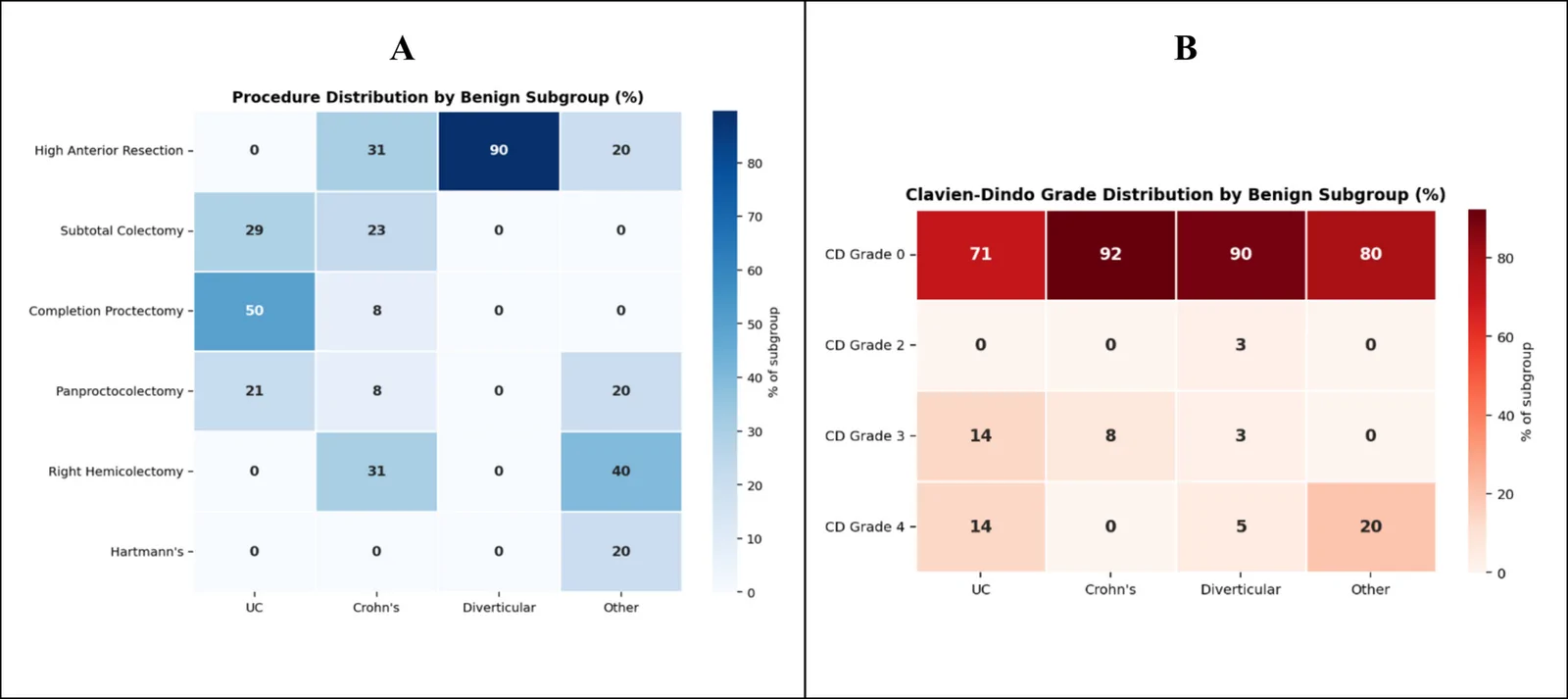
**A** Heatmap of procedure distribution across benign subgroups (% of each subgroup). **B** Heatmap of Clavien-Dindo (CD) complication severity by benign subgroup (% of each group)

The distribution of operative procedures differed significantly across subgroups (Table 3 and Fig. 3). Diverticular disease was managed almost exclusively by high anterior resection (90.0%), consistent with its anatomical predilection for the sigmoid colon. UC required predominantly extirpative procedures like completion proctectomy (50.0%), subtotal colectomy (28.6%), and panproctocolectomy (21.4%). Crohn’s disease showed the greatest procedural diversity high anterior resection (30.8%), right hemicolectomy (30.8%), and subtotal colectomy (23.1%), consistent with the heterogeneous anatomical distribution of Crohn’s pathology and the disease-specific principle of bowel conservation. Stoma formation rates differed significantly across subgroups (*p* < 0.001) and operative time was longer in UC (median 292 min) compared to Crohn’s (median 192 min). There were no statistically significant differences between the groups regarding individual postoperative complications or 30-day readmission rates (Fig. 3B). UC had the highest rates of CD Grade III (14%) and CD Grade IV (14%), reflecting the complexity of IBD surgery. Crohn’s disease and diverticular disease had predominantly uncomplicated courses (CD Grade 0: 92% and 90%).

**Table 3:** Operative procedures and perioperative outcomes by benign subgroup

Variable	UC (*n* = 14)	Crohn’s (*n* = 13)	Diverticular (*n* = 39)	Other (*n* = 5)	*p*-value
Procedures					< 0.001***
High anterior resection, *n* (%)	0 (0%)	4 (30.8%)	35 (89.7%)	1 (20%)	
Completion proctectomy, *n *(%)	7 (50.0%)	1 (7.7%)	0 (0%)	0 (0%)	
Subtotal colectomy, *n* (%)	4 (28.6%)	3 (23.1%)	0 (0%)	0 (0%)	
Panproctocolectomy, *n* (%)	3 (21.4%)	1 (7.7%)	0 (0%)	1 (20%)	
Right hemicolectomy, *n* (%)	0 (0%)	4 (30.8%)	0 (0%)	2 (40%)	
Stoma formed, *n* (%)	11 (78.6%)	5 (38.5%)	5 (12.8%)	2 (40%)	< 0.001***
Intraoperative					
Operative time, min median (IQR)	292 (228-343)	192 (180-380)	240 (193-282)	210 (190-218)	0.548
Estimated blood loss, mL median	50	50	50	50	0.958
Conversion to open, *n* (%)	2 (14.3%)	1 (7.7%)	8 (20.5%)	0 (0%)	0.515
Recovery					
LOS, days median (IQR)	6 (5-11)	6 (5-9)	5 (4-10)	10 (6-10)	0.466
Complications					
Any complication, *N* (%)	4 (28.6%)	2 (15.4%)	11 (28.2%)	1 (20.0%)	0.801
Anastomotic leak, *n* (%)	0 (0%)	0 (0%)	2 (5.1%)	1 (20%)	0.227
Intra-abdominal collection, *n* (%)	3 (21.4%)	2 (15.4%)	2 (5.1%)	1 (20%)	0.319
Prolonged ileus, *n* (%)	3 (21.4%)	0 (0%)	4 (10.3%)	1 (20%)	0.319
CD Grade III-IV, *n* (%)	4 (28.6%)	1 (7.7%)	3 (7.7%)	1 (20.0%)	0.203
30-day readmission, *n* (%)	1 (7.1%)	0 (0%)	0 (0%)	0 (0%)	0.248

*LOS* = length of stay; *CD* = Clavien–Dindo

***p < 0.001

Kruskal-Wallis *H* (continuous); chi-square or Fisher’s exact (categorical). “Other” subgroup (*n* = 5) reported descriptively only

## Discussion

Our study investigated perioperative outcomes in patients undergoing robotic surgery for benign colorectal resections at a large UK district general hospital. Using a comparator group of cancer resections performed during the same period, we evaluated the perioperative performance of the robotic platform in benign disease. Our findings demonstrate that while robotic surgery is safe and reproducible for benign conditions, these cases present distinct challenges. Most notably a higher conversion rate compared to cancer resections was noted in our cohort (15.5% vs. 6.9%, *p* = 0.031).

Patients in the benign cohort were generally 15 years younger and had fewer comorbidities; however, they were significantly more likely to have a history of PAS (33.8% vs. 19.6%, *p* = 0.012). Adhesiolysis in a previously operated abdomen distorts tissue planes and increases the risk of visceral injury, a challenge inherent to any surgical platform [23]. Within our benign cohort, diverticular disease yielded the highest subgroup conversion rate. This likely reflects the specific pathological complexity of advanced diverticulitis including the management of colovesical fistulae and inflammatory fixation rather than a limitation of the robotic system itself. In such instances, timely conversion represents clinical judgement rather than platform failure.

Despite the increased risk of conversion, the presence of PAS did not translate into inferior postoperative outcomes. There were no statistically significant differences in operative time, major complications (Clavien-Dindo III-IV), or length of stay between patients with and without a past surgical history. Furthermore, PAS did not increase readmission rates or blood loss. This suggests that the robotic platform’s technical advantages including 3D high-definition vision and wristed instrumentation may help mitigate the difficulties of a “hostile” abdomen. The wristed design allows for precise dissection with reduced shear forces, potentially minimising trauma to the abdominal wall and rectus sheath compared to traditional methods [22]. Although in some series have demonstrated that laparoscopic- and robotic-assisted surgery result in clinically equivalent return of bowel function, length of stay, postoperative pain, and opioid use [23]

Our results align with existing literature, where robotic conversion rates typically range from 5 to 15%. This is notably lower than some reported laparoscopic series, where PAS has been shown to raise conversion rates from 11.4 to 19.6% and quadruple the risk of inadvertent enterotomy [17, 24, 25]. In our series, the robotic system maintained stable visualization and consistent workflows even in technically demanding cases, such as restorative proctectomies, which were more frequently performed in the PAS group. This supports the growing consensus that robotic assistance is a safe and feasible option for complex benign disease [26, 27].

However, several limitations must be acknowledged. This was a retrospective, single-center study with inherent selection and information bias. The lack of a concurrent laparoscopic control arm prevents direct platform-specific comparisons. Furthermore, the study period was interrupted twice by the COVID-19 pandemic, during which elective robotic surgery was halted in favour of emergency and cancer prioritization. This may have impacted the learning curve, which was further complicated by varying surgeon volumes and the lack of a formal protocol for case assignment.

Finally, while the clinical benefits are evident, the higher costs associated with robotic systems remain a point of contention [21]. The “other” benign subgroup was too small for robust statistical analysis, and long-term functional outcomes such as urogenital function and quality of life were not captured. Future multicenter randomized controlled trials are required to determine if the intraoperative precision and ergonomic advantages of the robot justify the investment and training required for benign practice. Nevertheless, our data suggest that robotic surgery can be safely expanded to include benign colorectal disease, offering potential benefit to the younger, frequently reoperated IBD and diverticular populations.

## Conclusion

Robotic colorectal surgery is safe and reproducible across benign and malignant indications within specialised colorectal robotic centres. Complication profiles matched published benchmarks. Benign cases had a higher conversion rate, but this was driven by prior surgical burden and operative complexity, not platform limitation, and it did not increase morbidity. These data supports the expansion of robotic commissioning into benign practice.

## Data Availability

Fully anonymized datasets used and analyzed during the current project are available from the corresponding author upon reasonable request.
